# Applications of patient-derived tumor xenograft models and tumor organoids

**DOI:** 10.1186/s13045-019-0829-z

**Published:** 2020-01-07

**Authors:** Go J. Yoshida

**Affiliations:** 1 Department of Pathology and Oncology, Juntendo University School of Medicine, 2-1-1, Hongo, Bunkyo-ku, Tokyo, 113-8412 Japan; 2 Present Address: Department of Immunological Diagnosis, Juntendo University Graduate School of Medicine, 2-1-1, Hongo, Bunkyo-ku, Tokyo, 113-8412 Japan

**Keywords:** Acquired resistance, Avatar models, Carcinoma-associated fibroblasts, Co-clinical trials, Heterogeneity, Immunodeficient mice, Organoids, PDX models, Translational research, Tumor microenvironment

## Abstract

Patient-derived tumor xenografts (PDXs), in which tumor fragments surgically dissected from cancer patients are directly transplanted into immunodeficient mice, have emerged as a useful model for translational research aimed at facilitating precision medicine. PDX susceptibility to anti-cancer drugs is closely correlated with clinical data in patients, from whom PDX models have been derived. Accumulating evidence suggests that PDX models are highly effective in predicting the efficacy of both conventional and novel anti-cancer therapeutics. This also allows “co-clinical trials,” in which pre-clinical investigations in vivo and clinical trials could be performed in parallel or sequentially to assess drug efficacy in patients and PDXs. However, tumor heterogeneity present in PDX models and in the original tumor samples constitutes an obstacle for application of PDX models. Moreover, human stromal cells originally present in tumors dissected from patients are gradually replaced by host stromal cells as the xenograft grows. This replacement by murine stroma could preclude analysis of human tumor-stroma interactions, as some mouse stromal cytokines might not affect human carcinoma cells in PDX models. The present review highlights the biological and clinical significance of PDX models and three-dimensional patient-derived tumor organoid cultures of several kinds of solid tumors, such as those of the colon, pancreas, brain, breast, lung, skin, and ovary.

## Background

Since the first investigations to develop drugs using animal models of leukemia were reported in 1950 [1], many types of murine models transplanted with human tumors have been developed to predict responses to chemotherapy. Many human tumor models were generated in immunodeficient mice by subcutaneous or orthotopic injection of tumor cell lines that had been propagated in culture for several months to several decades. Although this model served as a gold standard, primarily because of ease of manipulation, tumor cell lines frequently acquired unanticipated phenotypes during adaptation to in vitro culture conditions that often differed between laboratories, resulting in minimal resemblance to the parental tumors. For example, immortalized cells cultured long-term in vitro exhibit changes in tumor hallmarks caused by genetic and epigenetic alterations. Thus, substantial limitations have precluded the application of conventional xenograft models for drug screening and estimating the pre-clinical efficacy of drugs [2].

To develop a model more likely to mimic human tumors, patient-derived tumor xenografts (PDXs) have been established as a useful tool for translational research [3–6]. Implantation of small pieces of tumors surgically dissected from cancer patients into highly immunodeficient mice allows tumor growth and subsequent transplantation into secondary recipient mice. PDXs often maintain the cellular and histopathological structures of the original tumors **(**Table 1**)** [7–9]. Furthermore, cytogenetic analysis of tumor cells from PDXs has revealed significant preservation of the genomic and gene expression profiles between PDXs and parental patient tumors [10–14]. Notably, sensitivity to standard chemotherapeutics in PDXs closely correlates with clinical data in patients from which the PDXs are derived [12, 13, 15–18]. All of these characteristics demonstrate that PDXs are more useful as predictive experimental models of therapeutic responses.

**Table 1:** Comparison between PDX models and organoids

	PDX	Organoids
Genetic/epigenetic alterations	Similar	Similar
Pathohistological characteristics	Similar	Similar
Response to anti-cancer drugs	Similar	Similar
Use of immunodeficient animals	Yes	No
Reliability as pre-clinical models	Yes	Yes
Quantity of cells for establishment	Large	Small

Tumor organoids are generated by three-dimensional culture of primary cancer cells and have a high success rate [19–25]. Organoid cultures closely recapitulate the morphological and genetic/epigenetic features of the parent tumors **(**Table 1**)** [20, 21, 25, 26]. Accumulating evidence reveals that primary cancer cells subjected to three-dimensional culture give rise to many different types of primary organoids, in which the heterogeneous composition of the original tumors is largely conserved [22, 27, 28]. This culture method provides promising opportunities to establish large biobanks with relevant clinical materials that can be used to perform drug screening and facilitate chemical discovery [24, 29]. Tumor organoids are superior to conventional models, as organoids constitute a minute incarnation of an in vivo organ, and thus can better recapitulate the characteristics of the parental tumor, even after many passages. Organoids have enormous potential for the identification of optimal treatment strategies in individual patients [28].

## PDX models maintain in vivo structure

A major advantage of PDX models in cancer research is the retention of the original tumor architecture. Although cancer cell lines are frequently transplanted into immunocompromised mice as a reproducible method to establish tumor tissues in vivo, the resultant tumors do not fully exhibit the distinct histopathological characteristics observed in clinical settings. One important consideration in this regard is that cells within tissues are surrounded by an extracellular matrix (ECM), a meshwork of proteins and proteoglycans consisting of laminin, collagen, and fibronectin, which regulates the integrity of epithelial structure formations. The ECM provides structural support, stability, flexibility, and shape to the tissue, and also mediates cell polarity, intracellular signaling, and cell migration [30, 31]. Intracellularly, ECM-induced signaling pathways are transmitted mainly through integrin molecules, which are heterodimeric transmembrane receptors that mediate cell adhesion to the ECM. Integrins act as bridges between the ECM and the internal cytoskeleton by transducing key intracellular signals through association with clusters of kinases and adaptor proteins in focal adhesion complexes [32]. These interactions are distinctly mediated by specific integrin heterodimer binding to individual ECM components [33]. The signals are transmitted via the cytoskeleton to nuclear transcription factors and ultimately change the gene expression profile [34]. As such, highly specific and localized signaling cascades can be activated by the ECM in association with various available growth factors through sequences of reactions involving networks of proteases and sulfatases [35, 36].

The tumor microenvironment comprises the ECM and stromal cells, including endothelial cells, pericytes, carcinoma-associated fibroblasts (CAFs), immune cells, and proinflammatory cells [37, 38]. One of the most prominent cell types in the tumor stroma is CAFs, which are activated fibroblasts. CAFs often exhibit an α-smooth muscle actin (α-SMA)-positive myofibroblastic phenotype, which is typical of fibroses and wound healing, and is a pro-inflammatory phenotype [36, 39]. Tumor-promoting CAFs influence tumor hallmarks to promote cancer growth, progression, and metastatic spread as well as remodeling of the ECM [36, 39, 40]. CAFs contribute to ECM remodeling by secreting TGF-β1, which is synthesized as an inactive multidomain complex, and is activated through multiple extracellular mechanisms that require integrins, proteases, and thrombospondin-1 [41, 42]. TGF-β1 has a potential role in the regulation of ECM remodeling during cancer progression. Exposing CAFs to TGF-β promotes anisotropic fiber organization and increased matrix stiffness through increased α-SMA expression and RhoA activation [43]. These changes in cellular contractility result in the CAF-mediated changes in the ECM architecture.

Tumors in the PDX model have similar pathohistological and genetic characteristics to the parent tumor, and exhibit similar susceptibility to anti-cancer therapies [7, 10, 11, 15, 16]. Therefore, PDX models based on grafting cancer tissue subcutaneously, orthotopically, or under kidney capsules in immunodeficient mice provide relevant pre-clinical cancer models. The mouse strains widely used for tumor formation and propagation are classified into (i) nude mice, which lack a thymus and are unable to produce T cells; (ii) non-obese diabetic severe combined immunodeficiency (NOD-SCID) and SCID-beige mice, which lack T and B cells; and (iii) NOD-SCID IL2R-γ null (NSG or NOG) mice, in which T, B, and NK cell activity is completely absent. Due to differential immunological impairments in these models, it is expected that more permissive mouse strains such as NOD-SCID, SCID, and NSG, will increase the efficiency of xenotransplantation over that of nude mice. In fact, a very low engraftment success rate (10–25%) was reported after transplanting tumor fragments of different pathohistological types into nude mice, whereas transplantation into NOD-SCID mice resulted in a higher engraftment rate (25-40%) for breast cancer, non-small cell lung cancer and malignant melanoma [44]. An extremely novel study evaluated the use of zebrafish for PDX, and reported that transparent zebrafish larvae allow for visualization of single tumor cells and their response to treatment, thereby providing a rapid screening platform [17].

However, it is important to note that human stromal cells are gradually replaced by murine counterparts after transplantation into immunodeficient mice, which suggests that implanted human cancer cells retain the potential to recruit murine stromal cells to their niche. Importantly, there are some differences between ligands secreted by human and murine fibroblasts. Human-derived interleukin (IL)-2 stimulates efficiently the proliferation of murine T cells, whereas mouse IL-2 stimulates human T cells with significantly lower efficiency [45, 46]. Furthermore, the T cell stimulating potential of IL-4 appears to be species-specific [45]. On the other hand, IL-15 binds to human or mouse IL-15 receptor α with equally high affinity [47], suggesting that human-derived IL-15 can function on murine cells. However, human NK cells are weakly sensitive to murine IL-15 [48]. In order to resolve the issues related to species specificity, co-implantation of human CAFs and tumor cell suspensions extracted from PDXs into secondary recipient mice could provide an optimal setting for evaluating human tumor cell-stroma cell interactions.

## Patient-derived tumor cell organoid culture mimic parental tumors

From a histopathological perspective, PDX models largely retain parent tumor architecture, as described above. Further, at the cellular level, both inter-tumoral and intra-tumoral heterogeneity, as well as the phenotypic and molecular characteristics of the original cancer are also preserved in PDX models [12, 49, 50]. From this perspective, human cancer tissues or tumor cells derived from PDX models that mimic the biological and molecular characteristics of the original cancer can be employed to provide clinically relevant donor cells for in vitro modeling. Tumor tissues from PDX models can be used to generate three-dimensional tissue explants in vitro or primary cell cultures on a petri dish. Use of PDXs has the advantage that the original tissue can be serially propagated in vivo, making additional materials available for repeated experiments and alleviating the limitations inherent to collecting multiple patient samples. To more fully represent the range of biological and molecular characteristics of clinical samples, a panel of established xenograft lines covering multiple cancer tissues of origin and subtypes is required for selection of the most appropriate model to address particular experimental questions.

Furthermore, mounting evidence suggests that organoids are three-dimensional constructs comprised of multiple cell types that originate from pluripotent stem cells by means of self-organization, and are capable of simulating the architecture and functionality of native tissues and organs [25, 51]. Organoids permit both in vitro and in vivo investigations, and represent one of the latest innovations in development of models to recapitulate the physiologic processes of whole organisms. Organoids have been successfully generated from primary tumors of the breast, colon, pancreas, and prostate [23, 25]. These tumor-derived organoids have emerged as pre-clinical models that have the potential to predict personalized response to treatment. For example, a living biobank of tumor organoids from patients with metastatic gastrointestinal cancer was demonstrated to recapitulate the therapeutic responses of these patients to anti-cancer agents in clinical trials [29].

## Use of PDX models for precision medicine

**Fig. 1:**
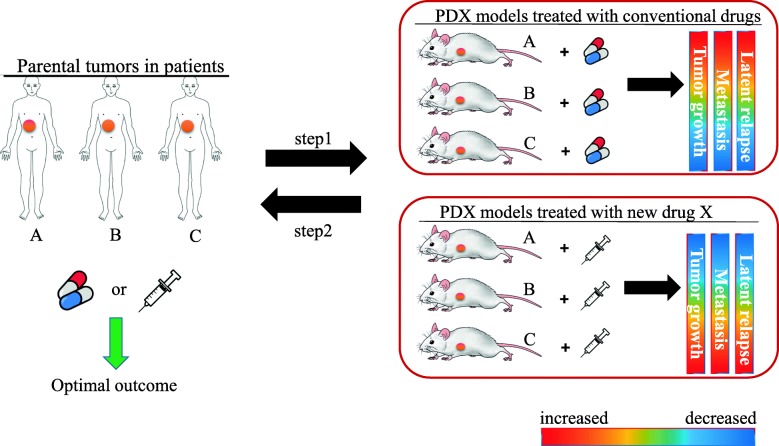
Identification of optimal therapeutics using PDX mouse clinical trials. PDX models are potentially useful when the optimal course of treatment cannot be readily determined for individual patients. For instance, in the illustration, there are three patients (A-C) with gastric cancer, who hope to receive treatment with the novel therapy drug X if its therapeutic efficacy is proven. In this case, it would be time-consuming and require significant clinical risk to compare the therapeutic response to conventional drugs and the new drug X without “co-clinical trials.” While xenografts derived from patient A respond to drug X, xenografts derived from patient C respond to conventional treatments, but not drug X (**step 1**). Contrastingly, patient B–derived xenografts partially respond to both therapeutics. This pre-clinical screening by an avatar model is helpful to determine which treatment would have the optimal outcome in each patient (**step 2**)

Mounting evidence demonstrates that PDX models have the potential to predict effectively the efficacy of both conventional and novel anti-cancer therapeutics, suggesting that these models could be employed in “co-clinical trials” [52]. Thus, investigations in vivo and in clinical trials could be performed in parallel in order to identify therapeutic target molecules, even in rare types of cancer. Particularly, the concept of “co-clinical trials” incorporates patient selection strategies based on molecular abnormalities or the identification of the machineries of resistance to anti-cancer agents, for the development of precision medicine aimed at personalizing anti-cancer treatment. In this context, PDX models can be also used as an “avatar model [53],” in which PDX models obtained from cancer patients enrolled in a clinical trial can be treated with the same therapy administered to the patient, thereby permitting identification of novel biomarkers of sensitivity or resistance to the anti-cancer treatment(s) of interest (Fig. 1).

### Colorectal adenocarcinoma

Acquired resistance of tumors to chemotherapeutics is a major reason for treatment failure [54, 55], and the introduction of novel drugs or combinational therapy permits selection of effective therapeutic strategies for second-line treatment. Based on this concept, Misale et al. treated advanced colorectal adenocarcinoma (CRC) patients with anti-EGFR antibodies as single agents after the initial response to EGFR inhibitors resulted in disease relapse due to emerging resistance [12]. Thus, Misale et al. investigated how acquisition of resistance to EGFR-targeted therapies can be reduced in CRC tumor cells using the PDX model. In vitro genetic screening and functional investigations identified that dual blockade of EGFR and MEK prevents acquired resistance, and Misale et al. performed experiments in vivo using PDXs derived from a CRC patient carrying a quadruple wild-type gene profile (*BRAF*, *KRAS*, *NRAS*, and *PIK3CA*), which recapitulates the expression profile of patients sensitive to anti-EGFR antibodies. While treatment of PDX models with the MEK inhibitor pimasertib alone only slightly reduced tumor growth, treatment with the EGFR inhibitor cetuximab effectively reduced cancer proliferation by more than 70% [12]. Notably, the subsequent regrowth of these tumors suggested resistance to drug re-challenge, which is similar to clinical findings. By contrast, combination treatment with cetuximab and pimasertib induced a complete response, in which tumor tissues remained undetectable for more than 6 months. These findings suggested that combination treatment is highly likely to inhibit development of resistant tumors with intra-tumoral heterogeneity, and highlighted the utility of PDX models in establishing highly effective treatment regimens.

**Fig. 2:**
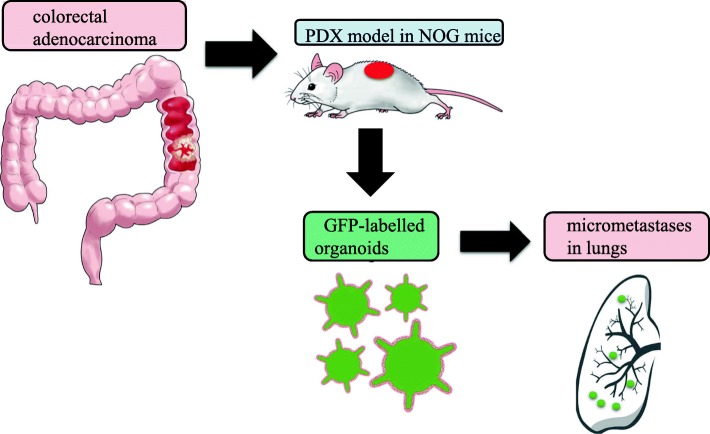
High-sensitivity detection of distant micrometastases of PDX-derived organoids by GFP transduction. After a primary colorectal adenocarcinoma (CRC) diagnosed as a moderately differentiated type was surgically resected, CRC cells were subcutaneously implanted into NOG mice to establish a PDX model. PDX tissue was treated with collagenase to obtain tumor cell suspension. CRC organoids were then established from PDX tissue and expanded in three-dimensional culture using the artificial extracellular matrix after infection with GFP lentivirus. These GFP-labelled organoids implanted orthotopically revealed distant micrometastases in the lungs within 3 months [56]

Okazawa et al. established green fluorescent protein (GFP)-labelled CRC PDX-derived organoids to detect spontaneous micrometastatic lesions [56]. Micrometastases, which are cancer cell depositions smaller than 2 mm, have often been overlooked in the pathohistological analysis of sections from affected distant organs in experimental murine models, because they may not be detected with this approach. Therefore, Okazawa et al. developed a protocol to efficiently transduce GFP lentivirus into PDX-derived CRC organoids in three-dimensional culture prior to transplantation, enabling highly sensitive detection of micrometastases in distant organs such as the liver and lungs (Fig. 2). Using this technology, Okazawa et al. employed a PDX model to demonstrate that lung micrometastases could be detected in the majority of engrafted mice 3 months after transplantation. Moreover, liver metastases were detected within 1 month after injection of organoids into the spleen. Further, the injection of CRC organoids into the rectal submucosa of immunocompromised mice resulted in metastatic dissemination into the lungs, but not into the liver, likely through the inferior vena cava.

### Pancreatic cancer

Although the progression of pancreatic ductal adenocarcinoma (PDAC) is driven by constitutive activation of RAS/RAF/MEK/ERK signaling, MEK1/2 inhibition with trametinib is not clinically effective in PDAC patients. A recent study revealed that trametinib-induced autophagy flux increases the survival of PDAC cells subjected to MEK1/2 inhibition [57]. Combination treatment with trametinib and chloroquine increased apoptotic cell death in a PDX model derived from a distant neck metastasis that was refractory to the conventional FOLFOX regimen. It is widely accepted that chloroquine obstructs autophagy flux, increasing ubiquitination, p62/SQSTM1 activation, and LC3-II accumulation [58]. Furthermore, the growth of orthotopic and/or subcutaneous PDX tumors in NOD/SCID mice was synergistically inhibited by combination treatment with trametinib and chloroquine [57]. The efficacy of this modality was also demonstrated in PDX models using gain-of-functional mutated *NRAS*-driven malignant melanoma and *BRAF*(*V600E*)-driven colorectal cancer. Notably, the therapeutic efficacy of this combination was superior to that of conventional anti-PDAC drugs such as gemcitabine. The compensatory activation of protective autophagy through the ULK1/AMPK/LKB1 axis appears to be responsible for susceptibility to trametinib in the presence of chloroquine.

### Brain tumors

Singh et al. reported that SOX2 expression is markedly elevated in PDX derived from glioblastoma multiforme (GBM) [59]. They established PDX models derived from 20 GBM patients, comprising different subtypes of putative GBM driver oncogenes such as *EGFR*, *MET*, and *PDGFRA*. Remarkably, all four IDH1 mutant PDX lines also exhibited high expression levels of SOX2. High SOX2 expression was present in GBM PDX that were driven by different oncogenes, suggesting that multiple oncogenic signaling pathways are likely to converge to drive expression of this pluripotency transcription factor. While PDX tumors maintained the putative oncogenic mutations of several receptor tyrosine kinases (RTKs) in the parental tumors, the significant increase in SOX2-expressing GBM cells may reflect the functional state of the subset of GBM cells capable of driving tumor growth in the mouse brain rather than genetic differences between the original patient tumor and PDX model. Singh et al. demonstrated that the transcriptional regulatory network comprised of SOX2, OLIG2, and ZEB1 is independent of upstream RTKs, and is capable of driving glioma initiating cells. Mithramycin is an antibiotic isolated from *Streptomyces plicatus* that binds to specific DNA regions to inhibit transcription of specific genes. Notably, mithramycin downregulated SOX2 and ZEB1 in sarcoma cells [60], and effectively inhibited growth of a SOX2-positive cell population that propagates medulloblastoma [61]. Administrating mithramycin in vivo markedly reduced expression of SOX2, OLIG2, and ZEB1, which coincided with dramatic reductions in tumor growth [59]. Taken together, these studies employing PDX models strongly suggest the importance evaluating the efficacy of combining mithramycin treatment with chemotherapy and radiation therapy in future investigations.

### Breast carcinoma

PDX models maintain the essential properties of the original patient tumors, including metastatic tropism, suggesting their physiological relevance for study of human cancer metastasis [4]. In immunodeficient mice, PDXs spontaneously metastasize many of the same organs affected in the original patient. In addition, mesenchymal stem cells (MSCs) in the PDX model enhance tumor growth rates by promoting angiogenesis, decreasing necrosis, and increasing blood volume, which would contribute to the observed increase in tumor growth. Lawason et al. demonstrated using the PDX model that progression to high metastatic burden is associated with increased proliferation and Myc expression, which can be attenuated by the treatment with cyclin-dependent kinase (CDK) inhibitors [8]. In this study, the most metastatic PDX had the highest percentage of cancer stem-like basal primary tumor cells, while the least metastatic PDX had the lowest. This suggests that primary tumors contain a rare subpopulation of stem-like cells, and that the relative abundance of these cells could correlate with metastatic potential. Thus, Lawason et al. used PDX models to propose a hierarchical model for metastasis, in which metastases are initiated by cancer stem-like cells, which proliferate and differentiate to produce advanced metastatic disease.

### Lung cancer

Chen et al. recently demonstrated an unexpected plasticity and interaction of lung squamous cancer cells (LSCCs) with the tumor microenvironment [62]. Overexpression of SOX2 in the TUM622 cell line, which was established from a PDX model, enhances spheroid-forming potential and drives a hyperplastic to dysplastic alteration in acinar phenotype, in which apical-basal cell polarity is disrupted, and solid non-invasive spheroids are formed. Remarkably, the presence of CAFs inhibits SOX2-induced dysplasia and restores an acinar-like phenotype, but TUM622 cells appear to exhibit epithelial-mesenchymal transition (EMT) at the invasive front towards CAFs, thereby forming “teardrop”-shaped structures [62, 63]. Indeed, CAF-secreted stromal cell-derived factor-1 (SDF-1) promoted EMT and the acquisition of stemness in LSCCs [64]. Although the majority of LSCCs were positive for E-cadherin and only a small population were positive for Vimentin and SOX2, these factors showed considerable heterogeneity in TUM622-derived spheroids [62]. Because there were cells positive for both E-cadherin and Vimentin, it is likely that partial EMT occurs in spheroids, the PDX model and the original tumor [62, 65].

Single cancer cell migration, also known as mesenchymal migration, is characterized by fibroblast-like morphology, but effective metastasis of cancer cells can occur without complete loss of epithelial morphology or complete acquisition of mesenchymal morphology. Cancer cells undergoing mesenchymal migration are enriched at the invasive front in vivo, consistent with previous findings that partial EMT is involved in collective tumor migration [65, 66]. Leader cells expressing mesenchymal-like or basal epithelial traits are located at the front of the follower epithelial cancer clusters, and drive their collective migration in response to microenvironmental cues. SOX2 appears to induce the commitment and differentiation of TUM622 cells to the squamous lineage instead of regulating epithelial/mesenchymal plasticity [62, 67]. SOX2 preferentially interacts with the transcription factor p63, as opposed to the transcription factor OCT4 in LSCCs, which is the preferred SOX2-binding partner in embryonic stem cells [68]. FGFR1 accelerates tumor development without forcing cells toward a particular tumor subtype. By contrast, SOX2 appears to be critical in driving cells toward an aggressive and penetrant LSCCs phenotype [67, 69]. Furthermore, CAF-derived CD81-positive exosomes mobilize Wnt11 produced by breast carcinoma cells, thereby activating β-catenin-independent Wnt planar cell polarity (PCP) signaling, which promotes lung metastasis [70]. Chen et al. suggested that the β-catenin-dependent canonical Wnt signaling pathway and cancer stem cell marker SOX2 synergistically induce acinar morphogenesis of TUM622 cells in three-dimensional culture [62]. Further investigations are warranted to determine how SOX2 overexpression and co-culture with CAFs affects the β-catenin-independent PCP pathway when spheroid cells derived from this PDX model of lung cancer acquire an invasive phenotype via partial EMT.

### Malignant melanoma

Both intrinsic and acquired therapy resistance remains a serious challenge for the management of *BRAF*(V600E)-mutant malignant melanoma. Many resistant cases exhibit reactivation of MAPK and PI3K signaling in the presence of MAPK-targeting agents. De novo lipogenesis is emerging as a central player in multiple oncogenic processes. Constitutive activation of the lipogenic pathway in tumor tissue is required for the synthesis of phospholipids, which function as essential building blocks of membranes and promote cell growth and proliferation. In vitro, a marked decrease in de novo lipogenesis was observed in all *BRAF*(V600E)-mutant therapy-sensitive, but not therapy-resistant, cell lines in the presence of the BRAF inhibitor vemurafenib [71]. Remarkably, BRAF inhibition induced only a moderate decrease in expression of sterol regulatory element-binding protein-1 (SREBP-1), a master regulator of lipid metabolism, and did not significantly affect lipogenesis in therapy-resistant melanoma cells. To assess the therapeutic potential of these findings, Talebi et al. investigated the impact of SREBP-1 inhibition in an in vivo pre-clinical *BRAF*(V600E)-mutant melanoma model. Talebi et al. selected a PDX that responded poorly to BRAF inhibitors alone [71, 72]. SREBP-1 contributes to the anti-tumor response induced by BRAF inhibition, and SREBP-1 inhibition sensitizes therapy-resistant melanoma cells to MAPK-targeting therapy. In vivo analysis of oxidative stress revealed that while fatostatin or vemurafenib treatment alone did not significantly increase lipid peroxidation, combined vemurafenib/fatostatin treatment greatly enhanced lipid peroxidation [71]. Notably, SREBP-1 inhibition enhances the sensitivity to BRAF inhibitors in a pre-clinical PDX model of melanoma [71].

### Ovarian cancer

Choi et al. established PDX models derived from serous adenocarcinoma and chemoresistant carcinosarcoma to investigate the therapeutic potential of itraconazole, an orally bioavailable anti-fungal drug that inhibits the enzyme lanosterol 14α-demethylase [73]. Previous reports identified itraconazole as a potent antagonist of the Hedgehog (Hh) signal pathway, which is different from the pathway involved in the inhibitory effect of this drug on fungal sterol biosynthesis [74]. Systemically administered itraconazole suppresses Hh pathway activity and medulloblastoma growth in a mouse allograft model similar to other Hh pathway antagonists, and surprisingly, itraconazole inhibits the Hh pathway at serum levels comparable with those of patients undergoing anti-fungal therapy [74, 75]. This is a typical example of drug repurposing, in which the anti-cancer properties of medications otherwise administered for non-malignant disorders serve to develop new treatments strategies for cancer [58]. Mechanistically, itraconazole antagonizes the Hh pathway component Smoothened (Smo) through a mechanism distinct from that of cyclopamine and other known Smo antagonists, and prevents ciliary accumulation of Smo mediated by Hh stimulation. Both the Hh and mammalian target of rapamycin (mTOR) signaling pathways are associated with angiogenesis in the tumor microenvironment. In ovarian cancer PDX models, addition of itraconazole to paclitaxel significantly enhanced therapeutic efficacy compared with paclitaxel monotherapy. Expression of CD31 and VEGFR2 (angiogenesis markers), Gli1 (hedgehog signaling downstream molecule), and S6K1 (mTOR pathway) were all decreased in tumors treated with paclitaxel and itraconazole combination therapy [73]. Itraconazole has synergistic effects when used in combination with paclitaxel-based chemotherapy, which is one of the most effective available chemotherapeutics for ovarian cancer. Table 2 provides the summary of the latest important papers using PDX models and organoids.

**Table 2:** Recent investigations of solid tumors using PDX models

Tumor type	Reference	Animal	Site	Metastasis
Colon adenocarcinoma	Misale et al. [12]	NOD-SCID mice	Subcutaneous	Liver
PDX models derived from a quadruple wild-type (*KRAS*, *NRAS*, *BRAF*, and *PIK3CA*) colorectal tumor are sensitive to EGFR blockade alone. EGFR/MEK combination blockade is superior to treatment with anti-EGFR antibodies, or to delivering a MEK inhibitor when resistance to cetuximab or panitumumab has already developed.				
Tumor type	Reference	Animal	Site	Metastasis
Colon adenocarcinoma	Fujii et al. [24]	NOG mice	Subrenal capsule and spleen	Liver
Fujii et al. established a colorectal tumor organoid library comprised of 55 organoids derived from 52 tumors and 43 patients, including hyperplastic polyps and sessile serrated adenoma/polyps. Pathohistological subtypes as well as differentiation hierarchies of CRCs were cell-intrinsically conserved regardless of environment (i.e., in patients, in vitro, and in PDX models). PDX models established by transplantation into the kidney subcapsules of immunocompromised mice developed the size of engrafted subrenal CRCs that secrete niche factors, including p38-MAPK, TGF-β, and EGF. In contrast to the robust engraftment efficiency in subrenal capsules, the metastatic capacity of spleen-injected CRC organoids was diverse.				
Tumor type	Reference	Animal	Site	Metastasis
Colon adenocarcinoma	Fior et al. [17]	Zebrafish	Subcutaneous	None
Fior et al. generated and treated five zebrafish-based PDX (zPDX) models of colon cancer derived from different patients, and treated zPDXs with the FOLFOX regimen over 3 days. Two PDXs responded to treatment, as indicated by increased caspase 3 cleavage. These two sensitive zPDXs corresponded to patients in whom CEA levels remained stable 6 months after surgery without relapse. Contrastingly, among the three zPDXs in which FOLFOX was not effective, the corresponding patients developed increasing CEA levels and clinical evidence of relapse. Furthermore, Fior et al. investigated the predictive effect of the EGFR inhibitor Cetuximab in combination with FOLFIRI regimen, finding that resistant zPDX models are derived from tumors with mutations in either *BRAF* or *KRAS*.				
Tumor type	Reference	Animal	Site	Metastasis
Colon adenocarcinoma	Okazawa et al. [56]	NOG mice	Orthotopic	Lungs
Pieces of resected CRCs were subcutaneously implanted into NOG mice to generate the PDX model. The organoid cells were then extracted from the PDX model for tissue culture, and CRC organoids were infected with GFP lentivirus, allowing highly sensitive visualization of micrometastases (Fig. 2). Notably, lung micrometastases were detected in three out of four mice examined 2.5 months after orthotopic injection of GFP-labelled PDX-derived organoids. The implantation of organoids into the rectal submucosa of NOG mice resulted in metastatic dissemination into the lungs, but not the liver, presumably through the inferior vena cava.				
Tumor type	Reference	Animal	Site	Metastasis
Pancreatic cancer	Zhou et al. [76]	Nude mice, SCID mice	Orthotopic	None
Because insulin growth factor 1 receptor (IGF1R) is highly expressed in both pancreatic cancer cells and stromal fibroblasts, Zhou et al. developed nanoparticles with recombinant human IGF1 conjugated to magnetic iron oxide carrying anthracycline doxorubicin (IGF1-IONP-Dox), and demonstrated an enhanced therapeutic effect compared with conventional Dox treatment in the orthotopic pancreatic ductal adenocarcinoma (PDAC) PDX model. T2-weighted magnetic resonance imaging (MRI) revealed systemic delivery of IGF1R-targeted Dox after administration of IGF1-IONP-Dox. Notably, non-specific uptake of IGF1-IONP-Dox in the spleen did not cause apoptosis, as demonstrated by lack of active caspase 3.				
Tumor type	Reference	Animal	Site	Metastasis
Pancreatic cancer	Witkiewicz et al. [77]	NSG mice	Subcutaneous	None
PDX models of PDAC enable precision therapy, as resistance to MEK inhibitors is paradoxically associated with compensatory Akt signaling activation [78]. Witkiewicz et al. used this model to demonstrate that combination therapy with trametinib (MEK inhibitor) and dasatinib (tyrosine kinase Src inhibitor) significantly suppresses PDX tumor proliferation. Furthermore, the Bcl-2 inhibitor ABT737 and checkpoint kinase inhibitor AZD7762 induces a therapeutic response in cooperation with conventional anti-cancer drugs such as docetaxel and gemcitabine.				
Tumor type	Reference	Animal	Site	Metastasis
Pancreatic cancer	Rajeshkumar et al. [79]	Nude mice	Subcutaneous	None
PDX models of PDAC respond more robustly to mitochondrial complex I inhibitors (phenformin and metformin) than to other metabolic inhibitors, including a glutaminase inhibitor, a transaminase inhibitor, and an autophagy inhibitor. Amino acids and metabolites involved in glycolysis such as lactate are decreased by complex I inhibitors, while oxidized glutathione is increased. There is no correlation between phenformin response and genetic abnormalities in *TP53*, *PTEN*, *SMAD4*, and *KRAS*. Although phenformin has not been applied clinically due to the risk of lactic acidosis [80], this study suggests the clinical promise of this biguanide agent.				
Tumor type	Reference	Animal	Site	Metastasis
Cholangiocarcinoma	Garcia et al. [14]	SCID mice	Subcutaneous	None
Five PDX models of cholangiocarcinoma exhibited identical *KRAS* mutations to those of the tumors from which they were derived. Indeed, the oncogenic *KRAS* mutation frequently occurs as a point mutation in codon 12, and these mutations result in constitutive activation of the PI3K pathway and RAS/RAF/MEK/ERK axis, which promote cancer cell survival and proliferation [81]. Cell-cycle regulatory proteins, including Chk1 and E2F1, were selectively down-regulated by BET protein inhibitor JQ1 in JQ1-sensitive PDX models. While JQ1 failed to affect c-Myc expression in JQ1-insensitive PDX models, a BET inhibitor down-regulated c-Myc in JQ1-sensitive PDX tumors, which was accompanied by down-regulation of downstream transcriptional targets such as Chk1 and E2F1.				
Tumor type	Reference	Animal	Site	Metastasis
Glioblastoma multiforme	Lee et al. [82]	Nude mice	Orthotopic	None
Lee at al. developed GBM recurrent PDX models induced by temozolomide, in which the immediate peak stabilization of HIF1α in PDX cells after exposure to chemotherapy is expected to represent an essential step in the conversion of non-cancer stem cells into undifferentiated cancer stem cells (CSCs). In both GBM6 (classical, MGMT hypermethylated), and GBM43 (proneural, MGMT unmethylated) PDX models, HIF1α levels were increased in the CD133-positive CSC population both post-therapy and at disease recurrence.				
Tumor type	Reference	Animal	Site	Metastasis
Glioblastoma multiforme	Singh et al. [59]	NOD-SCID mice	Orthotopic	None
Ectopic co-expression of SOX2, OLIG2, and ZEB1 transforms tumor-suppressor-deficient astrocytes into glioma-initiating cells in the absence of an upstream RTK oncogene. Among the three transcription factors, SOX2 expression is significantly upregulated in PDX GBMs. Histone H3 lysine 27 residue (H3K27) acetylation was present in more than 90% of the PDX SOX2 binding regions in the three analyzed patient GBM specimens, which indicates these regions as active *cis*-regulatory elements in patient GBM.				
Tumor type	Reference	Animal	Site	Metastasis
Glioma	Fack et al. [83]	NOD-SCID mice	Orthotopic	None
Fack et al. applied in situ metabolic profiling and LC-MS on brain sections of glioma PDX and human glioma samples with and without isocitrate dehydrogenase 1 (IDH1) mutations. Mass spectrometry imaging (MSI) and LC-MS analysis of orthotopic IDH-mutated glioma PDX models revealed IDH-specific adaptive mechanisms in metabolic pathways. Notably, cystathionine-β-synthase expression is a novel prognostic factor in the oligodendroglial glioma subtype.				
Tumor type	Reference	Animal	Site	Metastasis
Medulloblastoma	Garner et al. [84]	Nude mice	Orthotopic	None
Three group 3 medulloblastoma PDX models were established to evaluate the therapeutic efficacy of FTY720, an immunosuppressant that has currently been approved for treatment of multiple sclerosis [85]. FTY720 activates the tumor suppressor protein phosphatase 2A, and thus treatment of human medulloblastoma PDX cells with FTY720 arrested the cell cycle at the G1 phase and induced caspase-dependent apoptotic cell death.				
Tumor type	Reference	Animal	Site	Metastasis
Breast carcinoma	Lawson et al. [8]	NOD-SCID mice	Orthotopic	Lungs, bone marrow, liver, brain
In this study, the most metastatic PDX model (HCI-010) exhibited the highest percentage of basal/stem-like tumor cells, while the least metastatic model (HCI-002) had the lowest. This suggests that primary tumors contain a rare subpopulation of stem-like cells, and that the relative abundance of these cells correlates with metastatic potential. Higher-burden metastatic cells entered the cell cycle, expressing lower levels of quiescence and dormancy-associated genes and higher levels of cell-cycle-promoting genes, including CD24, CDK2, MMP1, and MYC, which have been associated with reactivation after dormancy. Dinaciclib, a CDK inhibitor that induces apoptosis in high MYC-expressing cancer cells via synthetic lethality [86, 87], significantly inhibits the metastatic potential of PDX models derived from drug-naïve patients.				
Tumor type	Reference	Animal	Site	Metastasis
Breast carcinoma	Evans et al. [13]	Nude mice	Orthotopic	None
More than 25 PDX models derived from triple-negative breast cancer (TNBC) tissues varied in the extent of PI3K and MAPK activation. PDXs were also heterogeneous in their sensitivity to chemotherapeutic agents; while PI3K, mTOR, and MEK inhibitors repressed growth but did not cause tumor regression, the PARP inhibitor talazoparib caused drastic regression in five of 12 PDXs. Notably, four out of five talazoparib-sensitive models did not harbor germline *BRCA1/2* mutations, but several had somatic alterations in homologous repair pathways, including *ATM* deletion and *BRCA2* alterations.				
Tumor type	Reference	Animal	Site	Metastasis
Breast carcinoma	Yu et al. [88]	NOD-SCID mice	Subcutaneous	None
To elucidate the mechanism of 5-Aza-2'-deoxycytidine (decitabine) action on TNBC, Yu et al. investigated DNA methyltransferases (DNMTs) expression levels, which were correlated with response to decitabine in breast cancer organoid models based on PDX tumors derived from TNBC patients recruited to a prospective neoadjuvant study of anthracycline- and taxane-based chemotherapy. Organoids and PDX models expressing elevated levels of DNMT proteins were more sensitive to decitabine than those expressing low levels of DNMTs, suggesting that DNMT may be a predictive biomarker for treatment response to decitabine in TNBC. This effect was mediated by decitabine-induced ubiquitination and lysosomal degradation of all three DNMTs, which was dependent on the E3 ligase TNF receptor-associated factor 6 (TRAF6). Decitabine treatment of PDX tissues also increased *TRAF6* transcription.				
Tumor type	Reference	Animal	Site	Metastasis
Breast carcinoma	Xiao et al. [9]	NSG mice	Subcutaneous	None
p21 protein–activated kinase 2 (PAK2) is activated by low C-terminal SRC kinase levels, which drives estrogen-independent tumor growth in patients with estrogen receptor (ER)–positive breast cancer resistant to endocrine therapy. Using a PDX model derived from ER(+)/PgR(+)/HER2(-) invasive ductal carcinoma, Xiao et al. confirmed that combination treatment with the PAK2 inhibitor FRAX597 and an ER antagonist synergistically suppressed breast tumor growth.				
Tumor type	Reference	Animal	Site	Metastasis
Breast carcinoma	Ikbale et al. [89]	NSG mice	Orthotopic	Lymph nodes, visceral metastasis
Compared with treatment-naïve TNBC–derived PDX, chemoresistant TNBC–derived PDX highly expressed Wnt10B-related molecules, including non-phosphorylated active β-catenin, Axin2, CD44, and HMGA2. ICG-001, a canonical Wnt signaling inhibitor, reduced tumor growth and lymph node metastatic burden. The combination of doxorubicin and ICG-001 efficiently repressed lung dissemination after tail vein injection of tumor cells that had dissociated from chemoresistant TNBC PDX. This synergistic effect was mediated by Bcl-2 downregulation.				
Tumor type	Reference	Animal	Site	Metastasis
Lung cancer	Weeden et al. [90]	NSG mice	Subcutaneous	None
PDX models derived from lung squamous cell carcinoma enabled Weeden et al. to identify *FGFR1* mRNA level rather than *FGFR1* amplification as a precise predictor of response to an FGFR tyrosine kinase inhibitor. Seventeen percent of PDXs evaluated (six out of 36 cases) exhibited *FGFR1* amplification, and FGFR inhibition decreased cell proliferation and enhanced differentiation, which decreased tumor growth rate and only modestly increased apoptotic cell death. Treatment with the FGFR inhibitor BGJ398 blocked both AKT and ERK signaling, and combination therapy with BGJ398 and a PI3K inhibitor was beneficial only in PDXs in which FGFR inhibition did not alter PI3K signaling.				
Tumor type	Reference	Animal	Site	Metastasis
Lung cancer	Drapkin et al. [91]	NSG mice	Subcutaneous	None
Because collection of circulating tumor cells (CTCs) enables non-invasive serial tumor sampling, Drapkin et al. established more than 30 PDX models of small cell lung cancer (SCLC) derived from not only biopsy tissues but also from CTCs. SCLC PDX models retain stable genome somatic alterations between initial PDX model generation and serial passages. Surprisingly, CTC-derived PDX models reflect the evolving chemotherapy sensitivities of the original tumor at the time of CTC collection. Etoposide resistance is positively correlated with activation of the Myc-associated gene signature, which is consistent with results obtained using the genetically engineered mouse model of SCLC [92, 93]. In comparison to other malignancies, SCLC PDX models exhibit increased clonal homogeneity and genomic stability.				
Tumor type	Reference	Animal	Site	Metastasis
Lung cancer	Gong et al. [94]	NOD-SCID mice	Subcutaneous	None
In general, patients with wild type EGFR (EGFRwt) do not respond to treatment with EGFR tyrosine kinase inhibitors (TKIs), while most patients with EGFR activating mutations initially respond to EGFR TKIs, but inevitably develop secondary resistance to TKI treatment. Simultaneous inhibition of EGFR and TNF prevents development of this acquired EGFR resistance. Notably, combination treatment with EGFR TKI (erlotinib) plus thalidomide was highly effective in inhibiting tumor growth in an EGFRwt PDX model, while EGFR inhibition or thalidomide alone was ineffective.				
Tumor type	Reference	Animal	Site	Metastasis
Lung cancer	Chen et al. [62]	Nude mice	Subcutaneous	None
The TUM622 cell line established from PDXs of lung squamous cell carcinoma has increased spheroid-forming capacity due to overexpression of the stem cell factor SOX2, and shows a hyperplastic to dysplastic change in its acinar phenotype, in which apical-basal cell polarity is disrupted. Wnt/β-catenin signaling and SOX2, which contribute to normal lung development, are involved in acinar morphogenesis of TUM622 cells in three-dimensional cultures. Chen et al. reported enhanced epithelial/mesenchymal plasticity of TUM622 cells after incorporating cancer-associated fibroblasts (CAFs) into a spheroid culture system, and expanding this system to model not only cancer cell ECM, but also cancer cell-CAF interactions during tumorigenesis. CAFs antagonized oncogenic SOX2 to restore the formation of luminal structures and promote invasion.				
Tumor type	Reference	Animal	Site	Metastasis
Malignant melanoma	Hirata et al. [95]	NSG mice	Subcutaneous	Lungs
Intravital imaging of *BRAF*-mutant melanoma cells containing an ERK/MAPK biosensor revealed how the tumor microenvironment affects responses to BRAF inhibition by PLX4720. Indeed, *BRAF*-mutant melanoma cells respond to PLX4720 heterogeneously, and BRAF inhibition activates CAFs, leading to FAK-dependent melanoma survival signaling. Fibronectin-rich matrices with 3-12 kPa elastic modulus are sufficient to induce PLX4720 tolerance. The PDX model revealed that co-inhibition of BRAF and FAK abolishes ERK reactivation in tumor stroma, leading to more effective control of *BRAF*-mutant melanoma.				
Tumor type	Reference	Animal	Site	Metastasis
Malignant melanoma	Krepler et al. [96]	NSG mice	Subcutaneous	Brain, lungs
To facilitate the advancement of pre-clinical in vivo modeling, Krepler et al. established more than 450 PDX models. Half (55%) of all analyzed samples were *BRAF* hotspot mutants, 20% were *NRAS* mutants, 7% were *NF1* mutants, 2% were *KIT* mutants, 1.4% were *GNAQ/GNA11* mutants, and 18% were WT, all of which were consistent with the TCGA database. The simultaneous inhibition of MDM2 and ERK is highly effective in PDXs refractory to BRAF inhibitors.				
Tumor type	Reference	Animal	Site	Metastasis
Malignant melanoma	Talebi et al. [71]	Nude mice	Subcutaneous	None
SREBP-1 protects vemurafenib-resistant melanoma cells from lipid peroxidation. Fatostatin treatment alone of PDX MEL006 inhibits tumor growth more potently than vemurafenib. Importantly, combined vemurafenib/fatostatin co-treatment has a greater anti-tumor effect than either monotherapy regimen.				
Tumor type	Reference	Animal	Site	Metastasis
Malignant melanoma	Einarsdottir et al. [97]	NOG mice	Subcutaneous	None
PDX models derived from metastatic melanoma were established to assess heterogeneous responses after treating tumors with karonudib, which inhibits the oxidized nucleotide-sanitizing enzyme MTH1 [98]. Comparison of the mutation profile between response groups revealed that karonudib has a cytotoxic effect in melanoma PDX models, irrespective of the mutation statuses of the most common driver genes in melanoma. Importantly, high expression of ABCB1 is a potential resistance biomarker.				
Tumor type	Reference	Animal	Site	Metastasis
Head and neck squamous cell carcinoma (HNSCC)	Grasset et al. [99]	NMRI-nude mice	Subcutaneous	None
The PDX model revealed that the LOX inhibitor BAPN attenuated ECM remodeling and tumor stiffness with collagen I bundles by reducing EGFR tyrosine kinase activity of cancer cells. The Ca^2+^ channel blockers phenylalkylamine verapamil and nondihydropyridine diltiazem, used for the treatment of hypertension and arrhythmia for decades, are effective in PDX models for the prevention of collective tumor cell invasion by significantly downregulating phosphorylated myosin light chain 2. Mechanistically, tumor-derived ECM stiffness and activated EGFR signaling enhances intracellular Ca^2+^ concentration mediated by the L-type Ca^2+^ channel Cav1.1 in squamous cancer cells.				
Tumor type	Reference	Animal	Site	Metastasis
Ovarian cancer	Liu et al. [100]	Nude mice, NSG mice	Intraperitoneal space	Peritoneal dissemination such as omentum, liver, pancreas, bowel, spleen and diaphragm
Liu et al. established PDX models of ovarian carcinoma derived from floating tumor cells in the ascites of irradiated nude mice, and ascites in established PDX models were then implanted intraperitoneally into NSG mice. Fresh ascites-derived tumor cells from these PDX tumor-bearing NSG mice were transfected with lentivirus encoding firefly luciferase and mCherry for bioluminescence imaging (BLI) of PDX models. Cohorts of NSG mice with luciferized PDX tumors were treated with carboplatin, or paclitaxel, either as a monotherapy or in combination, followed by weekly BLI measurements, the results of which positively correlated with those of plasma CA125 and cell-free DNA assays. Notably, PDX models derived from platinum-refractory patients demonstrated significant resistance to carboplatin.				
Tumor type	Reference	Animal	Site	Metastasis
Ovarian cancer	Kim et al. [101]	NSG mice	Orthotopic	None
Kim et al. investigated the therapeutic effects of a PARP inhibitor (PARPi; olaparib), an ATR inhibitor (ATRi; AZD6738), and a CHK1 inhibitor (CHK1i; MK8776) for the treatment of *BRCA*-mutant high-grade serous ovarian cancer (HGSOC) cells. Although PARPi and CHKi as single agents modestly suppressed tumor growth, the addition of ATRi/CHK1i to PARPi in *BRCA*-mutant ovarian PDX models significantly decreased tumor volume compared with single-agent therapies. Remarkably, significant differences were observed in responsiveness to the PARPi-ATRi and PARPi-CHK1i combinations. While PARPi-CHK1i combination treatment significantly increased PDX tumor suppression over single-agent treatments, PARPi-ATRi combination treatment significantly increased the incidence of PDX tumor regression.				
Tumor type	Reference	Animal	Site	Metastasis
Ovarian cancer	George et al. [18]	NSG mice	Orthotopic	Diaphragm
PDX models derived from *BRCA*-mutant HGSOCs pathologically exhibited solid, pseudoendometrioid, and transitional cell carcinomas, referred to as SET features. Because approximately 50% of HGSOC patients have defects in homologous recombination (i.e., loss-of-functional mutations of *BRCA1/2*) [102], blocking cell cycle checkpoints could potentially induce synthetic lethality in HGSOC. Indeed, PDX models of *BRCA2*-mutant HGSOC exhibited higher levels of phosphorylated CHK1 than *BRCA*-intact HGSOC. PET imaging of PARP-1 with the PARP inhibitor analogue [^18^F] FTT was performed in PDX models, which revealed that PARP inhibitor treatment resulted in tumor suppression but not complete tumor regression, similar to the response observed in clinical settings.				
Tumor type	Reference	Animal	Site	Metastasis
Ovarian cancer	Choi et al. [73]	Nude mice	Subrenal capsule	None
Choi et al. examined the therapeutic effect of the antifungal itraconazole in PDX models of serous adenocarcinoma and carcinosarcoma. Combination therapy with paclitaxel and itraconazole significantly inhibited tumor growth and suppressed angiogenesis. Combination therapy was more effective in decreasing CD31, VEGF receptor, Gli1-mediated Hh, and mTOR signaling than paclitaxel monotherapy.				
Tumor type	Reference	Animal	Site	Metastasis
Ovarian cancer	Kondrashova et al. (2018) [49]	NOG mice	Subcutaneous and orthotopic	None
The response of PDX models to the PARP inhibitor rucaparib largely depends on *BRCA1* promoter methylation zygosity. PDX models were derived from 12 HGSOC patients, ten of whom were chemotherapy-naïve, and two of whom had received multiple lines of therapy. A heterozygous therapeutic response to rucaparib was observed among *BRCA1/2*-mutant HGSOC PDX models. Kondrashova et al. observed altered zygosity of *BRCA1* methylation, characterized by homozygosity in the chemo-naïve patient-derived samples and heterozygosity in the previously treated HGSOC patient–derived samples. Loss of homozygous *BRCA1* methylation is likely responsible in part for acquired rucaparib resistance.				
Tumor type	Reference	Animal	Site	Metastasis
Bladder cancer	Lee et al. (2018) [50]	NOG mice	Orthotopic	Muscle invasion
Lee et al. developed an optimized methodology to convert bladder tumor organoid lines into orthotopic PDX models with up to 80% efficiency using ultrasound-guided implantation of organoids between the bladder urothelium and lamina propria. Tumor evolution is common in organoids, even in the absence of drug treatment. Patient-derived bladder cancer organoids maintained heterogeneity, leading to clonal evolution. Notably, the basal organoid phenotype reversibly changed into the luminal phenotype in PDX models, likely due to cellular plasticity. PDX models retain drug responses to trametinib (MEK inhibitor) and gemcitabine.				

## Future challenges of PDX models

While the incorporation of PDX models in cancer research brings some exciting improvements, PDXs have important limitations that must be addressed to improve the availability of PDX models for translational research and pre-clinical investigations, including “co-clinical trials.” Issues that must be addressed or standardized to facilitate wide use of PDX models include (a) the site for implantation of original tumor fragments, (b) time course for PDX tumor tissue generation, (c) the engraftment rate, (d) replacement of human stroma with murine stroma, (e) failure to evaluate the immune system, and (f) the challenging problems of Matrigel.

### Optimal implantation site

It is important to define the best engraftment site (subcutaneous, subrenal capsule, or orthotopic implantation) in each tumor type of interest. Most of the published studies using PDX models have relied on surgical specimens, which naturally provide large quantities of tumor tissues. Although much effort has been expended in establishing PDX models of cholangiocarcinoma and head and neck squamous cell carcinoma, acquiring a sufficient volume for transplantation is prohibitive due to the small size of the original tumor. Thus, suitable methods with smaller samples obtained by biopsy or fine-needle aspirations should be established.

### Time course of PDX tumor tissue generation

Delay between the engraftment period in mice and optimal treatment schedules for patients is a limiting factor for the use of PDX models in real-time personalized medicine applications. Developing a PDX model for preclinical study generally requires 4–8 months with several tumor passages, which is much longer than patients can ordinarily wait to commence treatment. The second- and third-generation PDX passages require only 10 days to form a palpable xenograft. Due to the time requirement to generate PDX models, some groups are using short-term single-cell suspension and short-term culture in organoid models to evaluate sensitivity to potential treatments.

### Tumor engraftment rate

The engraftment failure rate is still high for some cancer types and phenotypes, which is a major obstacle to widespread PDX use. It is thus essential to improve tumor engraftment rates to an acceptable level, up to 60–70%. Most importantly, patients with breast and renal cancer whose tumors successfully engraft have the worst prognosis [4, 5, 103], which strongly suggests a selective pressure towards more aggressive phenotypes. It is possible that as the passages of the PDX model proceed, proliferative, and highly metastatic clones are selected to establish the next generation of PDXs.

### Replacement of human stroma with murine stroma

Intratumoral heterogeneity can be influenced by tumor-extrinsic factors in the microenvironment, including murine host cells. After 3–5 passages, when PDX models can be used for drug screening, tumor-associated stroma are almost completely replaced by murine-derived ECM and fibroblasts. This new murine stroma is likely to cause drastic changes in paracrine regulation of the tumor as well as in physical properties such as interstitial fluid pressure, which disturb drug distribution [104]. Importantly, some cytokines are species-specific. As stated previously, human-derived IL-2 stimulates the proliferation of murine T cells at similar concentrations, whereas mouse IL-2 stimulates human T cells at a significantly lower efficacy [45]. Furthermore, the T cell stimulating potential of IL-4 seems to be species specific. IL-15 binds to human or mouse IL-15 receptor α with equal high affinity [47]. Surely human-derived IL-15 can function on murine cells; human NK cells are poorly sensitive to murine IL-15 [48].

### Failure to evaluate the immune system

One key requirement in establishing PDX models is the need to use immunodeficient host strains for tumor engraftment and propagation. These mice lack functional elements of the immune system, so that foreign tumor tissues cannot be rejected. For example, NSG mice lack natural killer cells and both B and T lymphoid cells. Therefore, the contribution of the host immune system to responses to conventional chemotherapeutics cannot be assessed. Currently available PDX models are also unable to accurately assess immunotherapies such as vaccines and immune modulators, and drugs that activate the anti-tumor immune system.

### Disadvantages of Matrigel

Cellular interactions with the ECM profoundly alter not only gene expression pattern but also tumor cell behavior [105]. Tight ECM regulation in the tumor microenvironment is lost during engraftment, and tissue architecture degrades as PDX model tissue undergoes passages. Furthermore, Matrigel is frequently used to increase the engraftment rate in PDX models, since the presence of growth factors in Matrigel can favor engraftment of primary tumor cells. However, it should be recognized that this is a murine basement membrane extract [106], and Matrigel from murine sources can be a source of infectious murine viruses. In fact, treatment of primary cancer cells with lactate dehydrogenase-elevating virus-contaminated Matrigel in early studies may account for the reported instances of contamination, and for this reason, some groups produced their own Matrigel in order to avoid potential viral contamination [107].

## Conclusions

PDX models mimic not only the pathohistological and genetic/epigenetic features of original tumor tissues but also therapeutic responses to anti-cancer treatments. Therefore, PDX models have the potential to predict individual responses to drugs and treatments, thereby facilitating personalized medicine. Furthermore, many of the mechanisms underlying acquired drug resistance in many tumor types have been elucidated using PDX models. Although the tumor stroma, including CAFs, is replaced by mouse stroma during xenograft passages, the heterogeneity of the tumor microenvironment and the metastatic potential of the tumor cells are maintained in both subcutaneous and orthotopic PDX models. Notably, biofluorescence imaging of PDX models derived from organoids is a highly sensitive method for detecting micrometastatic lesions. Further investigations are necessary to develop strategies to evaluate the effects of immune-checkpoint inhibitors, because PDX models can only be established in immunocompromised mouse strains.

## Data Availability

The datasets used and/or analyzed during the current study are available from the corresponding author on reasonable request.

## Abbreviations

CAFs: Cancer-associated fibroblasts
CRC: Colorectal adenocarcinoma
CTC: Circulating tumor cell
ECM: Extracellular matrix
EMT: Epithelial-mesenchymal transition
GBM: Glioblastoma multiforme
GFP: Green fluorescent protein
HGSOC: High-grade serous ovarian cancer
Hh: Hedgehog
IDH: Isocitrate dehydrogenase
mTOR: Mammalian target of rapamycin
PDAC: Pancreatic ductal adenocarcinoma
PDXs: Patient-derived xenografts
RTKs: Receptor tyrosine kinases
SREBP-1: Sterol regulatory element-binding protein-1
TNBC: Triple-negative breast cancer
